# Prenatal psychological distress, access to mental health care and pathways between risk/protective factors and maternal postnatal depressive symptoms in the E.L.F.E. french birth cohort

**DOI:** 10.1192/j.eurpsy.2024.646

**Published:** 2024-08-27

**Authors:** A.-L. Sutter-Dallay, M. Bales, M.-A. Charles, V. D. W. Judith

**Affiliations:** ^1^Perinatal psychiatry, Charles Perrens Hospital and Bordeaux University; ^2^Perinatal psychiatry, Charles Perrens Hospital, Bordeaux; ^3^ Joint Unit ELFE, INSERM INED EFS; ^4^Social epidemiology team, Sorbonne University and INSERM U 1136 Pierre Louis-epidemiology and public health, Paris, France

## Abstract

**Introduction:**

Mental health of pregnant and post-partum women is sensitive to environmental factors. However, access to mental healthcare remains difficult, while little is known about protective factors nor about interactions between different exposures.

**Objectives:**

To explore on a large sample of women from the general population (i) the environmental and pregnancy characteristics independently associated with prenatal psychological distress and access to mental health care during pregnancy (ii) pathways between maternal, infant and parenthood vulnerabilities or risk/protective factors and postnatal depressive symtoms (PNDS) at 2 months post-partum (PP)

**Methods:**

The data from the French ELFE birth cohort were used. Available information about prenatal psychological status, access to mental health care and vulnerabilities-risk/protective factors for PNDS were collected during the maternity ward stay and at 2 months PP. PNDS were evaluated with the Edinburgh Postnatal Depression Scale (EPDS) at 2 months. Maternal/pregnancy characteristics independently associated with prenatal psychological distress and access to mental health care were explored using multivariate analyses. Pathways between risk/protective factors and PNDS at 2 months were investigated through Structural Equation Modeling.

**Results:**

Of the 15,143 mothers explored in the prenatal part of the study, 12.6% reported psychological distress (PPD), 25% had a prenatal consultation with a mental health specialist, 11% used psychotropic drugs of which 4% had no specialist follow-up. Decreased likelihood to consult a mental health specialist was found in young women, with intermediate educational level and born abroad. PPD was more frequent in women with very low economic status, alcohol/tobacco use, unplanned pregnancy, late pregnancy declaration, multiple and complicated pregnancy. In the postnatal part of the study (n=11,583) partner’s perceived antenatal emotional support, consultation with a mental health specialist before pregnancy, financial difficulties, prenatal psychological distress and experience of pregnancy were directly associated with the severity of maternal PNDS at 2 months PP, as well as perceived postnatal support, infant’s self-regulation skills, maternal ability to understand infant crying and infant hospitalisation.

**Conclusions:**

Perinatal professional support should begin antenatally and target the couple’s prenatal functioning, with particular attention to women presenting history of psychiatric disorders, especially when of low socioeconomic status. After delivery, addressing infant and parenthood characteristics is recommended.

**Disclosure of Interest:**

None Declared

